# Revealing Low Amplitude Signals of Neuroendocrine Cells through Disordered Silicon Nanowires‐Based Microelectrode Array

**DOI:** 10.1002/advs.202301925

**Published:** 2023-06-25

**Authors:** Francesco Maita, Luca Maiolo, Ivano Lucarini, Josè Ignacio Del Rio De Vicente, Antonio Sciortino, Mario Ledda, Valentina Mussi, Antonella Lisi, Annalisa Convertino

**Affiliations:** ^1^ Institute for Microelectronics and Microsystems National Research Council Via Fosso del Cavaliere 100 Rome 00133 Italy; ^2^ Institute of Translational Pharmacology National Research Council Via Fosso del Cavaliere 100 Rome 00133 Italy

**Keywords:** disordered silicon nanowires, electrical activity recording, microelectrode arrays, neuroendocrine cells

## Abstract

Today, the key methodology to study in vitro or in vivo electrical activity in a population of electrogenic cells, under physiological or pathological conditions, is by using microelectrode array (MEA). While significant efforts have been devoted to develop nanostructured MEAs for improving the electrophysiological investigation in neurons and cardiomyocytes, data on the recording of the electrical activity from neuroendocrine cells with MEA technology are scarce owing to their weaker electrical signals. Disordered silicon nanowires (SiNWs) for developing a MEA that, combined with a customized acquisition board, successfully capture the electrical signals generated by the corticotrope AtT‐20 cells as a function of the extracellular calcium (Ca^2+^) concentration are reported. The recorded signals show a shape that clearly resembles the action potential waveform by suggesting a natural membrane penetration of the SiNWs. Additionally, the generation of synchronous signals observed under high Ca^2+^ content indicates the occurrence of a collective behavior in the AtT‐20 cell population. This study extends the usefulness of MEA technology to the investigation of the electrical communication in cells of the pituitary gland, crucial in controlling several essential human functions, and provides new perspectives in recording with MEA the electrical activity of excitable cells.

## Graphical abstract

Based on disordered silicon nanowires, a newly designed microelectrode array (MEA) enables to record the low amplitude signals generated by a population of neuroendocrine cells of the pituitary gland. The method here provides an effective tool for exploring the complexity of the neuroendocrine system and new perspectives for MEA technology.

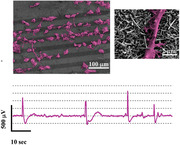

## 1 Introduction

Unraveling the cellular communication across coordinated electrogenic cell networks, by including neurons, cardiomyocytes, or endocrine cells, is crucial to gain a deeper understanding of the brain activity, heartbeat, or hormone secretion, and develop more sensitive diagnostic tools and efficient disease treatments.

Over the last decades, intracellular patch clamp^[^ ^1^, ^2^, ^3^ ^]^ has become the “gold standard” in studying in vivo and in vitro single cells, by obtaining information of the entire electrophysiological spectrum including action potentials (APs), subthreshold potentials, membrane oscillations, and their underlying ionic currents. Anyway, patch clamping is unsuitable to capture the global activity of a wide electrogenic cells network across a range of biological spatial and temporal scale due to its invasiveness and sensitivity, limited to record currents in individual cells for a short time. By contrast, extracellular electrophysiological recording methods, such as the ones that use microelectrode arrays (MEAs), are rising as promising tools for long‐term analysis studying cellular circuit‐connectivity, physiology and pathology under in vitro or in vivo condition.^[^ ^4^, ^5^, ^6^, ^7^, ^8^ ^]^ An array of single‐contactable electrodes records the electrical field potentials induced by the ion flux across the cell membranes^[^ ^9^, ^10^ ^]^ without disturbing the metabolic events and damaging the cell membrane. Thus, that approach allows long‐term recordings as well as the possibility to perturb the electrical activity of the electrogenic cell networks at specific sites using electrical stimulation. These features are pivotal to understand the communication modes in cell groups, regions, or micro‐organs and, combined to the novel fabrication strategies of integrating MEAs in flexible and soft electronic devices,^[^ ^11^, ^12^, ^13^ ^]^ open the way to new and powerful diagnostic, therapeutic or prosthetic tools in diverse medical fields.

Recent progresses in materials science and nanotechnology have enabled to overcome important shortcomings of standard MEA technique and provide novel performing MEAs by modifying the sensing electrode surface through micro‐ and nano‐structured patterning.^[^ ^5^, ^14^, ^15^, ^16^ ^]^ The micro‐nanostructured electrodes are indeed characterized by an increase of the effective sensing surface area, which leads to a decrease of the electrode impedance, thus allowing the usage of spatial high‐density recording tools. Furthermore, a tight cell–electrode coupling is ensured by the cells that closely engulf the nanosized MEA protrusions, thus minimizing the gap between cell and electrode resulting in a high seal resistance. Accordingly, these two key characteristics enhance the signal‐to‐noise ratio during measurement, by allowing to capture weak extracellular signals of the cells. While successful applications of the micro‐nanostructured MEAs have been reported for recording the electrical signals generated by neurons or cardiomyocytes,^[^ ^17^, ^18^, ^19^, ^20^, ^21^, ^22^, ^23^, ^24^, ^25^, ^26^ ^]^ the neuroendocrine cells have remained poorly investigated with the existing MEA approaches owing to their far smaller signal amplitudes as compared to neurons or heart cells.^[^ ^27^, ^28^ ^]^ In addition most of the reported micro‐nanostructured MEAs are characterized by regularly organized structures, such as sharp nanopillars,^[^ ^17^, ^18^, ^19^, ^20^, ^21^ ^]^ gold micro‐mushrooms,^[^ ^22^, ^23^ ^]^ plasmonic electrode arrays,^[^ ^24^ ^]^ or exotic structures like as nanopatterned volcano‐shaped MEA^[^ ^25^ ^]^ that require complex and expensive fabrication technologies impacting on the scalability of devices and reproducibility of the results across the labs. In this regard, randomly organized nanostructures with non‐uniform size distribution are gaining significant attention as suitable interface for cellular cultures.^[^ ^29^, ^30^, ^31^ ^]^ Specifically, disordered silicon nanowires (SiNWs) offer unprecedented versatility in terms of mammalian cell interfacing^[^ ^29^, ^31^, ^32^ ^]^ due to their architecture mimicking the extracellular matrix, and as probes of cellular function and biomolecules through electrical and optical signal transduction.^[^ ^31^, ^33^, ^34^, ^35^, ^36^, ^37^ ^]^ Moreover, these systems can be easily obtained by using low‐cost, high‐yield and facile bottom‐up fabrication methods at low temperature (<350 °C)^[^ ^35^, ^38^ ^]^ thus permitting their direct growth on specific flexible substrates like as polyimide.^[^ ^39^ ^]^

Here, we leverage the remarkable properties of the disordered SiNWs to capture in vitro the weak extracellular electrical signals of pituitary corticotrope AtT‐20/D‐16v cells. Specifically, we designed and fabricated a MEA with 12‐channels consisting of disordered SiNWs, covered by a thin film of gold (Au/SiNWs). The Au/SiNWs‐based MEA (NW_MEA), integrated in a customized recording system, allowed us to record in vitro for the first time, to the best of our knowledge, the electrical signals generated by a network of AtT‐20 cells under high extracellular calcium (Ca^2+^) concentrations.

The AtT‐20 cells, originally derived from mouse anterior pituitary tumor tissue, are a cellular model generally used for neuroendocrine studies, peptide secretion, and glucocorticoid regulation.^[^ ^40^ ^]^ They are excitable, possess voltage‐activated Ca^2+^ channels and express several neuronal features.^[^ ^41^ ^]^ We note that recording the electrical signals generated by a population of these cells through MEA approach is a relevant result. Indeed the pituitary gland is populated by five types of secretory cells (corticotrope cells are one type) generating low frequency spontaneous APs whose characteristics, in particular frequency and intensity of the spikes, depend on the concentration of extracellular Ca^2+^.^[^ ^42^, ^43^, ^44^, ^45^ ^]^ It is supposed that this spontaneous, intrinsic signaling of the pituitary cells plays a role in basal hormone release from these cells, which are organized into tightly wired large‐scale networks^[^ ^46^, ^47^, ^48^ ^]^ communicating with each other in both homotypic and heterotypic manner.^[^ ^49^, ^50^ ^]^ By considering that the role of the pituitary gland is crucial for essential human functions, including growth, reproduction, and our response to emotional and physical stress, the recording of the electrical activity in AtT‐20 cell network is a first step toward the exploration of the complex pituitary cells’ communication path. Additionally, since we note that the AtT‐20 cells constitutively express several neuronal features^[^ ^41^ ^]^ and in particular they can extend neurite‐like processes containing neurofilament (NF) proteins, the AtT‐20 cell network on NW_MEA can be also utilized as suitable neural model for extracellular electrophysiological experiments.

## 2 Experimental Section

### 2.1 Fabrication of SiNWs and NW_MEA

**Figure 1:**
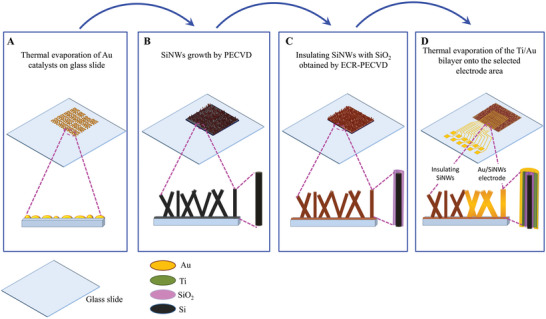
Schematic of the workflow of the NW_MEA fabrication steps: A) evaporation of Au layer, 2 nm thick, onto glass slide; B) VLS growth of SiNWs by PECVD; C) SiO_2_layer, 50 nm thick, obtained by ECR‐PECVD; D) creation of the electrodes and connecting pads by evaporating a Ti (20 nm)/Au (150 nm) bilayer onto the selected electrode area of the insulating SiNWs mat.

The workflow of the fabrication processes is summarized in **Figure** **1**. Au‐catalyzed SiNWs were initially produced by plasma enhanced chemical vapor deposition (PECVD) on microscope glass slides via vapor liquid solid (VLS) mechanism. In particular, to induce the NW growth, a 2 nm thick Au layer was selectively deposited onto a specific area of 1 × 1.2 cm^2^ (Figure 1A) of a glass slide obtained as follows: i) squared opening 1 × 1.2 cm^2^ was patterned into a negative photoresist layer, spun on the glass slide, by standard UV‐photolithography; ii) the thin Au layer was then deposited by thermal evaporation; iii) a final wet etching to remove the resist residue was performed by using acetone for 2 min. The substrate was successively transferred into the PECVD growth chamber and heated up to the temperature of 350 °C in N_2_ atmosphere at the pressure of 1 Torr. The growth of SiNWs (Figure 1B) was performed with SiH_4_ and H_2_ as precursors at a total pressure of 1 Torr. The flow ratio SiH_4_/(H_2_+SiH_4_) was fixed to 1:10 with SiH_4_ and H_2_ flow rates of 10 and 90 sccm, respectively.

A 13.6 MHz radio frequency with power fixed at 5 W was used to ignite the plasma. The growth time was fixed at 7 min. To ensure a proper electrical insulation among channels, a SiO_2_ layer (50 nm thick) was then deposited by electron cyclotron resonance (ECR)‐PECVD (Figure 1C) at room temperature, starting from a gas mixture of O_2_, SiH_4_, and He.^[^ ^51^ ^]^ The insulating film was deposited at a working pressure of 4 × 10^−3^ mbar and at microwave power of 700 W. Since the ECR‐PECVD was a conformal coating technique, it was reasonable to expect the individual NWs uniformly coated by the SiO_2_ film. Successively, a Ti (20 nm)/Au (150 nm) bilayer was evaporated to produce the 12 Au/SiNWs‐based electrodes and connecting tracks, obtained after a lift‐off process (Figure 1D) by using a positive potassium iodide solution diluted in de‐ionized water and processed at room temperature for 2 min to pattern the Au layer. The underlying Ti film was etched in a 5% HF solution for 5 s, followed by cleaning of the polymer residue from the electrodes and pads with acetone. The SiO_2_ coating of the SiNWs mat prevented current paths across different Au/SiNWs electrodes and was not affected by the Ti removal (the etching time with HF was too small). The role of the thin layer of Ti was solely devoted to improve the adhesion between the Au layer and the substrate.

### 2.2 Electrochemical Recording Platform

The NW_MEA, with the silicone well chamber sealed on top, was housed in a custom aluminum recording box. The box mounted an electronic interface board used a spring contact array to achieve both non‐destructive and reliable connections between the electrodes and the digital acquisition (DAQ) system. The DAQ system, composed by a commercial mother + child development board based on Intan Technologies neural chip RHD2216, was used to perform signal acquisition by acting as an interface between the measurement system and a PC software for data storage and visualization (Intan RHX). The system could perform 16‐bit acquisition on 16 channels at 30 000 sample/sec per channel. Moreover, digital band pass filter could be set with lower cut‐off frequencies in the range 0.1–500 Hz and upper cut‐off frequencies in the range 100 Hz–20 kHz to limit noise interferences and for offset removal. The chip was designed for intracortical or ECoG recording and had a typical low input‐referred noise of 2.4 µVrms. The board was provided with a co‐axial connector to enable the use of an external reference electrode with the minimum injection of noise in the system. An external platinum wire was bonded to the core of the co‐axial electrode and used as reference during the measurements. Every recorded voltage shown throughout this article was the electrode voltage, which was measured directly at the instrument (negligible Ohmic losses). All the measurements with cells were carried out at 37 °C by placing the recording box on a heating plate and controlling the temperature on the sample with a thermocouple.

### 2.3 Electrochemical Characterization of the NW_MEA and Planar MEAs

The NW_MEA and the planar MEAs, consisting of 12 planar gold and platinum electrodes (Au_MEA and Pt_MEA, respectively) having the same size and features of the nanostructured ones, with the silicone culture chamber were housed inside the recording board to perform the device characterization without cells by filling the culture chamber with Dulbecco's phosphate‐buffered saline (DPBS) solution. External reference electrode of platinum was connected to the interface board and the digital filters of the DAQ system were set to *f*_CUT‐LOW_ = 1 Hz and *f*_CUT‐HIGH_ = 7.6 kHz. Notch filter for 50 Hz removal was disabled to evaluate the immunity of the system to the line noise. A two‐point impedance measurement was performed for all electrodes between the electrode itself and the external reference electrode at four different frequencies (30, 100, 1000, and 5000 Hz) with 10 mVrms input signal amplitude. The impedance measurements were done with both the DAQ system embedded in the electronic board and a potentiostat VersaSTATs 4 by PAR. The measured impedance was compared by obtaining an excellent agreement in the range of frequency where the two systems overlap. Indeed, with the potentiostat it spanned a large frequency range from 0.1 Hz to 1 MHz. The impedance measurements were performed at room temperature in a Faraday cage at DC 0 V and with an AC signal of 10 mV. The tests were performed in DPBS, anyway different solutions (e.g., NaCl solution [0.9%] in de‐ionized and distilled water, and KCl 1 mm) were also tested to evaluate the stability of the electrodes. The solution was added and waited for 15 min before starting the measurement session, consisting of a minimum of four repetitions on the same electrode over a period time of 1 h.

### 2.4 Cell Culture

AtT‐20 D16V cells (American Type Culture Collection, Rockville, MD, USA) were grown in Dulbecco's modified Eagle's Medium (Euroclone), supplemented with 10% heat‐inactivated fetal bovine serum (Euroclone), 2 mm l‐glutamine (Sigma), 1.0 unit mL^−1^ penicillin (Sigma), and 1.0 mg mL^−1^ streptomycin (Sigma). The cells were grown in plastic dishes at 37 °C in a humidified incubator at 5% of CO_2_. Before the use, the Au/SiNWs substrates and MEAs were immersed in ethanol 70% for 30 min and washed by phosphate buffered saline (PBS). AtT‐20 D16V cells (10^5^ cells cm^−2^) were seeded both on the Au/SiNWs mat, NW_MEA, planar MEAs as well plastic Petri dishes, used as reference samples, and then cultured for up to 2 days.

### 2.5 Cell Viability, Cytoskeleton, and mRNA Expression Analysis

AtT‐20 cells viability was studied by a metabolic activity assay based on colorimetric water‐soluble tetrazolium salts oxidation test (Cell Proliferation Reagent WST‐1; Roche Diagnostics). The cells were seeded on both the Au/SiNWs mat and the plastic Petri dishes at a concentration of 3 × 10^4^ cells cm^−2^, were cultured for up to 4 days in a humidified incubator (37 °C, 5% CO_2_), and analyzed daily. At day 1, 2, 3, and 4, WST‐1 chemical (1:10) was diluted in the medium of AtT‐20 cells and maintained for 2 h in incubator, the cell supernatants (100 µL) were transferred in a 96‐well plate and the formazan dye was analyzed. The produced formazan dye was quantified by absorbance measurement at 450 nm with a scanning multiwell spectrophotometer (Biotrack II; Amersham Biosciences, Little Chalfont, UK). Cell viability was reported as the percentage of the absorbance of cells grown on Au/SiNWs mat in relation to the absorbance of those grown the plastic Petri dishes.

The AtT‐20 cells seeded both on the Au/SiNWs substrates and plastic Petri dishes, cultured for up to 4 days in a humidified incubator (37 °C, 5% CO_2_), were analyzed by actin fluorescence staining. Cells were first washed in PBS, fixed in 4% paraformaldehyde for 15 min, washed three times with PBS, and incubated with phalloidin tetrametylrhodamine isothiocyanate conjugated (1:500), an anti‐actin toxin (Sigma), for 1 h. After, the AtT‐20 cells were washed three times with PBS, counterstained for nuclei localization with Hoechst 33342 (trihydrochloride–trihydrate) and examined. The Au/SiNWs substrate was turned upside down on cover glasses and assessed by direct fluorescence for the actin filament visualization. Fluorescence measurements were obtained using an inverted microscope (Olympus IX51, RT Slider SPOT – Diagnostic instruments, Sterling Heights, MI, USA), equipped with a 10× and 20× objective and with a cooled CCD camera (Spot RT Slider; Diagnostic Instruments).

Total RNA was obtained from the AtT‐20 cells cultured both on the Au/SiNWs substrates and plastic Petri dishes for 3 days, using TRIzol Reagent (Invitrogen). 1 µg of total RNA was retrotranscribed using iScriptTM cDNA synthesis kit (Bio‐Rad). RT‐qPCR was used for the quantification of studied mRNA by SsoAdvanced Universal SYBR Green Supermix (Bio‐Rad) and Bio‐Rad Real‐Time PCR Detection Systems. cDNA templates (0.5 µL) were run in triplicate in 20 µL of volume using with 250 nm of specific primers. The examined mRNA markers were neurofilament heavy chain (NFH), class III beta‐tubulin (TuJ1), and *β*‐actin. Cycling parameters and qPCR protocol were reported in Ledda et al.^[^ ^52^ ^]^ The data were analyzed by following the method reported by Livak.^[^ ^53^ ^]^

### 2.6 Extracellular Recordings with NW_MEA and Planar MEA

NW_MEA, Au_MEA, and Pt_MEA were used to acquire extracellular signals from AtT‐20 cells. The devices were cleaned using ethanol and AtT‐20 cells (5 × 10^4^ cells cm^−2^) were seeded on the MEAs, and then cultured for up to 2 days to reach the monolayer confluence. The MEAs with the cell culture in HEPES buffer without calcium (HEPES 7.5 mm, MgSO_4_ 2 mm, KH_2_PO_4_ 1.25 mm, C_6_H_12_O_6_ 10 mm, NaCl 156 mm) were placed in the aluminum box slot and connected with the recording board. After having closed the box to limit the noise interference, the platinum reference electrode was inserted in the solution through an ad‐hoc hole. Hence, the electrical reliability of the contacts was verified through a preliminary impedance analysis at 1 kHz. Finally, the acquisition software was started selecting the following parameters: amplifier bandwidth of 1–7500 Hz, sampling rate = 30 000 sample/sec. The electrical activity was recorded by increasing concentration of the extracellular Ca^2+^ from 0 mm up to 50 mm(2, 10, 20, 30, 35, 40, 45, and 50 mm each 4 min, resulting in a recording period long 36 min). The different concentration of extracellular Ca^2+^ was obtained by adding, with micropipette, proper volume dilution (µL) of Ca^2+^ stock solution (1 m), in HEPES buffer inside the box (100 µL) for each concentration (mm) used.

### 2.7 Scanning Electron Microscopy

The morphology of the Au/SiNWs mat and NW_MEA was verified by a field emission scanning electron microscopy (SEM, ZEISS SIGMA 300) at an accelerating voltage of 5 kV. The size of the NWs was determined by combined measurements from top and cross section views.

To observe the immobilized cells onto the substrates, the NW_MEA and planar MEAs, the cells were fixed with 4% of paraformaldehyde. The hydrated cells were then coated by an evaporated thin (10 nm) Au layer. The observations were performed at an accelerating voltage of 5 kV.

### 2.8 Statistical Analysis

Data were acquired at 30 kHz ch^−1^ in binary format to ensure enough data compression and guarantee long stream acquisitions of 12 channels signals. Then the data were elaborated by using National Instruments Diadem 2019 software. At first, fast Fourier transform was applied to verify the harmonic content of data and residual line noise. Then, a second order Bessel low‐pass digital infinite impulse response filter was applied to data (*f*_CUT‐OFF_ = 40 Hz) to eliminate such residual contribution. After these preliminary operations, a significative content was verified at low frequencies (<10 Hz). For this reason, to limit occurrence of false positives during peak‐analysis routines, it was decided to operate a smoothing over mean value using a window of 50 points (1.6 ms of data at 30 kHz s^−1^). Hence, it was proceeded with analysis of peak positions and intensities, using as constraints for the algorithm a threshold value of 20 µV, that was three times the root mean squared (RMS) value (7.2 µV) and an interval width of 0.3 s for duration. Finally, the obtained data were further checked, and the spurious spikes and residual noise were discarded.

Unless otherwise stated, all electrochemical data were reported as mean ± SD, related box‐plots showed the interquartile range (IQR) as box, whiskers denoted the area within 1.5 IQR, the central mark showed the mean.

## 3 Results and Discussion

### 3.1 Fabrication of the NW_MEA on Microscope Glass Slide

**Figure 2:**
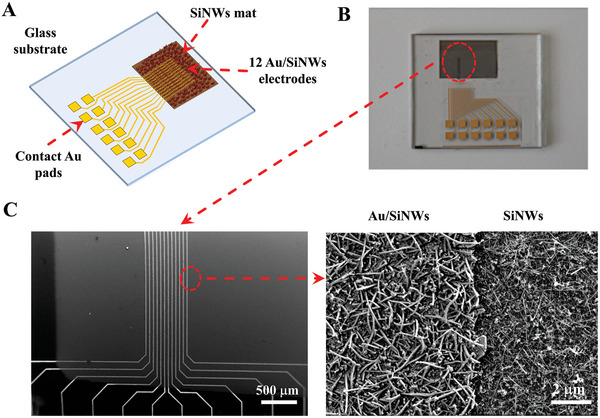
A) Schematic representation of NW_MEA on microscope glass slide: 12 Au/SiNWs‐based electrodes embedded in a SiNWs mat and connected with a 2×6matrix of Au pads for the electrical connections to the acquisition system. B) Photograph of a representative completed MEA. C) SEM images of the area with the electrodes array: the bright stripes are the 12 Au/SiNWs‐based electrodes. In the right panel a detail of the border conductive/insulating NWs.

The layout of the NW_MEA used here is illustrated in the **Figure** **2**A. 12 recording channels consist of a finger shaped area with a mat of SiNWs, covered by a thin film of Au (Au/SiNWs electrodes) and embedded in a mat of insulating SiNWs. The electrodes are 1 cm long and 25 µm wide, with an electrode spacing of 25 µm, and are connected to the lower 2 × 6 matrix of Au pads used to electrically connect the MEA to the acquisition system.

The SiNWs were fabricated by using PECVD onto a specific area (1 cm x 1.2 cm) of a microscope glass slide via VLS mechanism. An insulating film of SiO_2_ (50 nm) covered the SiNWs. Successively, 12 Au/SiNWs‐based electrodes and connecting tracks were obtained by thermal evaporation of a Ti (20 nm)/Au (150 nm) bilayer onto the patterned area (further details in the Experimental Section).

Figure 2B shows the picture of a representative fabricated device: the dark area corresponds to the mat of SiNWs and the Au/SiNWs‐based electrodes are inside the red highlighted inset. The SEM images in Figure 2C provide the top view of the electrode array. At higher magnification (right panel in Figure 2C) we can observe a dense ensemble of disordered and randomly oriented NWs, where the bright NWs are the metal electrodes. The pristine SiNWs are long around 2–3 µm with a tapered shape and an average diameter at the bottom of about 50–80 nm. After evaporating the Ti/Au bilayer, the Au/SiNWs have a more cylindrical shape and an increased average radial size, which is around 120–180 nm at the bottom.

Here we used a MEA design different from the traditional one, which typically consists of rounded or squared shaped electrodes with a size order of magnitude smaller. The strategy behind the proposed design is to develop a device with a uniform nanotopography, that is, without significant topographical discontinuity between the conductive areas of the electrodes and the surrounding insulating regions. We observe, indeed, that the mechanical interactions between micro/nano‐structured surfaces and cells greatly influence the cellular adhesion, proliferation, morphology, functionality, spreading, alignment, and fate.^[^ ^54^ ^]^ The proposed NW_MEA ensures the AtT‐20 cells to sense the same physical features, which allow the cells to express a uniform response, across the whole MEA surface. Because of the length of the electrodes, the measured signal will be the sum of the contributions of the cells adhering on each electrode, whereas the electrode width and spacing allow possible bridging of cells processes across the electrode array by permitting to address ensembles of cells, lying on different electrodes, that engage into cooperative activity or generate collective events.

### 3.2 Electronic Board for Data Acquisition

Electrogenic cells generate APs with low intensity amplitude. Neurons and cardiomyocytes depolarize to values of about +40 mV, while in neuroendocrine cells the AP peak generally does not overshoot 0 mV.^[^ ^27^, ^28^, ^43^, ^55^, ^56^ ^]^ As a consequence the AP amplitudes for neurons and cardiomyocytes are about 100 mV and 120 mV respectively, whereas for neuroendocrine cells about 80–60 mV. By considering that the corresponding extracellular field potentials are typically attenuated by a factor ranging from 1/100 to 1/1000, the extracellular signals to be recorded vary from hundreds to tens of microns.^[^ ^5^, ^57^ ^]^ Reading such low voltages requires also a performing acquisition electronics able to leverage the full capabilities of the nanostructured MEAs to achieve high signal‐to‐noise ratio while allowing a good flexibility and reliability of the measurement process.

**Figure 3:**
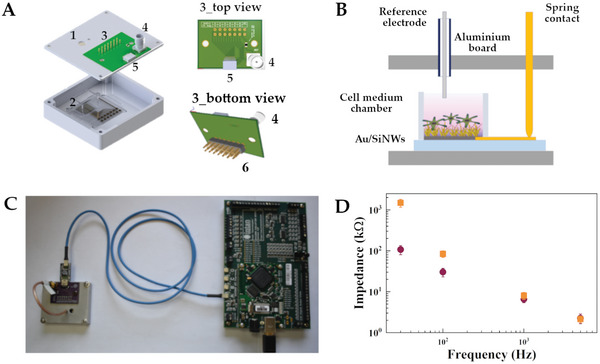
A) Schematic representation of the acquisition chamber consisting of an aluminum box (1) with the stage for the MEA accommodation and culture chamber (2). The box mounts on the top the electronic interface board (3) consisting of a 2 × 6 matrix of spring contacts (6), the reference connector (4) and the Intan RHD2216 connector (5). B) Setup used for recording the electrical signals generated by the AtT‐20 cellular population cultured on the electrodes of the NW_MEA device. C) Photograph of the complete platform depicting the customized recording board connected to the Intan RHD2216 board. D) Average electrochemical impedance of the Au/SiNWs (red symbols) and planar Au electrodes (yellow symbols) at the frequencies of 30, 100, 1000, and 5000 Hz (N = 36). The value of the standard deviations is indicated by the error bars. The impedances were measured with the setup in (C).

We therefore developed a customized recording platform that provided the proper electrical insulation, reliable electrical contacts, and low‐impedance interface. **Figure** **3**A depicts the layout of the developed recording chamber, consisting of an aluminum box (1) that allowed both electrical insulation from the external environment and electrical connections between the electrodes and the electronic acquisition board. For the in vitro experiments, the final MEAs were placed inside the box and the cells were cultured inside a silicone chamber sealed onto the MEA (2). The electronic interface board (3) was mounted onto the aluminum box with a 2 × 6 matrix of spring contacts (3_top view and 3_bottom view) which guaranteed both non‐destructive and reliable connections between the Au pads and the DAQ system. The board was also provided by a co‐axial connector (4) that enabled the use of an external reference electrode. DAQ system, composed by a commercial mother + child development board based on Intan Technologies neural chip RHD2216, performed data acquisition by acting also as an interface between the measurement system and a PC software used for data storage and visualization. The schematic recording configuration is summarized in Figure 3B, and the photograph of the experimental setup is in Figure 3C. This recording setup was used to assess the uniformity of the electrochemical impedance across the whole NW_MEA. Two‐point impedance measurements were performed at four different frequencies (30, 100, 1000, and 5000 Hz) by soaking the device with DPBS solution. To understand the impact of the NWs upon the electrical characteristics, we compared the electrochemical impedance of Au/SiNWs electrodes with that of reference planar Au electrodes. Specifically, we fabricated the planar MEA (Au_MEA) on microscope glass slide, consisting of 12 planar Au electrodes with the same size and features of the nanostructured ones, and performed the electrochemical characterization for the nanostructured and planar electrodes in the very same experimental conditions. To evaluate also the process uniformity, we analyzed three NW_MEAs of different batches and averaged the impedance over 36 individual electrodes, that is, by considering 12 individual channels for three different NW_MEA. Figure 3D shows the mean electrochemical impedance for Au/SiNWs (red symbols) and planar Au electrodes (yellow symbols) at the frequencies of 30, 100, 1000, and 5000 Hz. As expected, the surface nanostructuring significantly reduces the impedance of the NW‐based electrodes with respect to the planar ones because of an increased effective area. In addition, the low deviations (smaller than 25% for the lower tested frequencies) suggest uniformity throughout the electrodes of the same MEA, as well as a good repeatability of the fabrication process.

### 3.3 Biocompatibility Study of AtT‐20 Cells Cultured on Au/SiNWs Mat

We first studied the influence of the Au/SiNWs on ATt‐20 cells’ adhesion, morphology and viability through phase‐contrast microscopy and cytoskeletal actin filaments (F‐actin) staining analysis.

**Figure 4:**
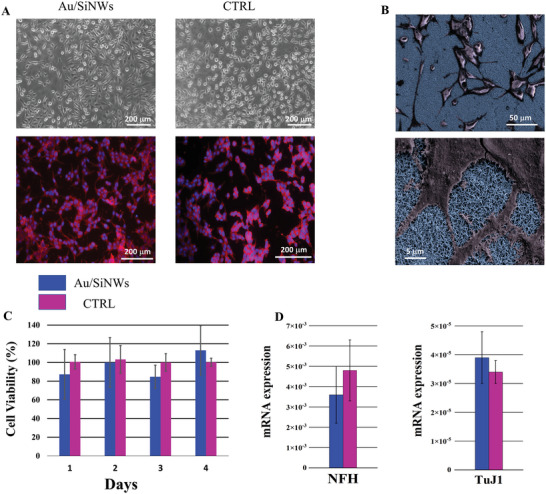
A) Phase contrast images (top panels) and fluorescence micrographs of DAPI/phalloidin staining (bottom panels) of AtT‐20 cells after 2 days of culture on both Au/SiNWs mat and the control Petri dish (CTRL). B) SEM images, at different magnification, of fixed AtT‐20 cells on Au/SiNWs mat. C) Cellular viability analysis of the AtT‐20 cells cultured on both Au/SiNWs mat (blue histograms) and the control Petri dish (pink histograms). D) mRNA study of the neuronal markers NFH and TuJ1for the AtT‐20 cells cultured 3 days on both the Au/SiNWs (blue histograms) mat and the control Petri dish (pink histograms).

Actin is a highly conserved protein that changes its spatial distribution during normal cell processes and in response to physical and chemical stimuli^[^ ^58^, ^59^, ^60^ ^]^ by controlling cells’ shape and internal organization. The cells were cultured for 2 days on both Au/SiNWs mat and a Petri dish, that was used as control experiment (CTRL), without any adhesion factor. The comparison between the phase contrast images (**Figure** **4**A, top panels) suggests that the nanostructured substrate did not induce a cytoskeletal filament rearrangement, as well as cellular morphology and adhesion changes. The cells, indeed, appeared well attached on the Au/SiNWs by maintaining their typically polygonal shape. The phalloidin fluorescent staining (red) study confirmed these results (Figure 4A, bottom panels) and highlighted that the cytoskeletal organization of the AtT‐20 cells cultured on Au/SiNWs mat was unaffected compared to the CTRL one. In Figure 4B, SEM images clearly displayed the presence of neurite‐like prolongations, several tens of microns long, as well as the formation of an extended cellular network.

WST‐1 test, a colorimetric assay for the quantification of cell metabolic activity based on oxidation of tetrazolium salts, was performed to study the viability of the ATt‐20 cells cultured on both the Au/SiNWs mat and the Petri dish (CTRL), 1, 2, 3, and 4 days.

The formazan quantification was performed by measuring the absorbance at 450 nm with an ELISA reader. The plot in Figure 4C compares the percentage of the absorbance of AtT‐20 cells cultured on Au/SiNWs mat (blue histograms) in relation to the absorbance of the CTRL cells (pink histograms), set to 100%, by indicating a comparable viability (about 85–100%) up to 4 days. The Au/SiNWs biocompatibility was further confirmed at the mRNA level by investigating via qRT‐PCR the transcripts expression of the neuronal markers such as the NFH and the TuJ1, genes physiologically expressed in the AtT‐20 cells. NFs are classified as intermediate filaments specific to neurons^[^ ^59^ ^]^ and together with microtubules and microfilaments, they form the neuronal cytoskeleton. On the other hand, TuJ1 is an early differentiation marker of neurons in the central and peripheral nervous systems. The mRNA expression analysis showed no statistically significant differences between these mRNA markers on cells cultured for 3 days on both Au/SiNWs mat and Petri dish. Collectively these results indicate that the nanostructured platform can support ATt‐20 cell adhesion, metabolic activity, cell viability, and neuronal commitment by providing evidence of its biocompatibility and cell‐friendly response.

### 3.4 Recording Electrical Activity Generated by AtT‐20 Cells with NW_MEA

Under unstimulated conditions, AtT‐20 cells show spontaneous extracellular Ca^2+^ dependent APs that have been recorded on single cells using perforated patch clamp technique.^[^ ^43^, ^62^ ^]^ It has been found that the raising of the external Ca^2+^ concentration increases the AP frequency and amplitude while APs are abolished for external Ca^2+^ concentration below the normal value (2 mm). Based on these findings, we recorded the electrical activity of *n* = 6 AtT‐20 cellular cultures on NW_MEAs by tuning the concentration of extracellular Ca^2+^ from 0 mm (HEPES buffer without Ca^2+^) up to 50 mm.

The recordings were divided into sequential periods of 4 min, characterized by an increased concentration of the extracellular Ca^2+^, by covering a total period of 36 min. Preliminary cell viability tests demonstrated that the investigated external Ca^2+^ concentration range did not show cytotoxicity toward the AtT‐20 cell populations over the recording period (Figure S1, Supporting Information). These results are consistent with the study performed on rabbit bone cells demonstrating that apoptotic changes can be observed after 8 h exposure to high extracellular calcium.^[^ ^62^ ^]^

**Figure 5:**
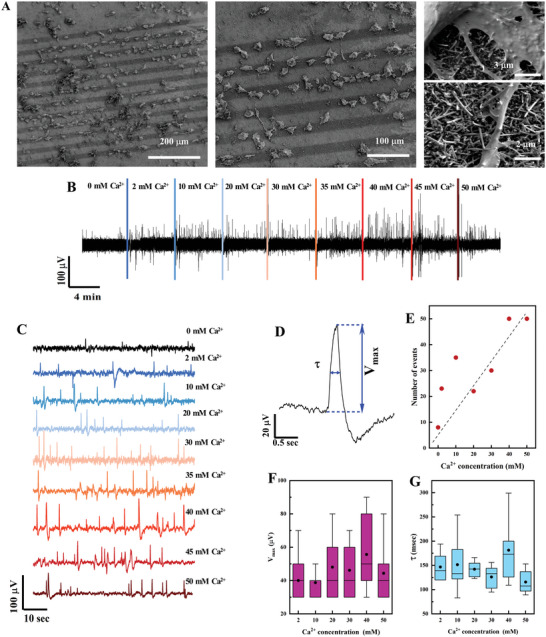
A) SEM images, at different magnifications, of AtT‐20 cells cultured onto the 12 electrodes of the NW_MEA. B) An overview of whole interval recording trace from a representative Au/SiNWs electrode with cells. The recording consists of sequential periods of 4 min, characterized by increased Ca^2+^ concentration in the HEPES solution. The colored lines indicate the time of Ca^2+^ addition. C) Recording segments of the trace in (B) for different Ca^2+^ concentrations and selected at 2 min after the Ca^2+^ addition. D) A representative spike. V_max_ indicates the amplitude of the spike and τ the duration at 50% of V_max_. E) Number of events recorded during the whole activity shown in (B) as a function of the Ca^2+^ content. Events with V_max_ > 20 µV were considered. F) V_max_ and G) τ distribution of the spikes recorded at different concentration of Ca^2+^. Half of the data points are within the boxes; bottom and top whiskers are computed as 1.5 interquartile range of the lower and upper quartile, respectively. Mean and median values of the distributions are shown as black dots and solid lines.

The AtT‐20 cells’ coverage of the electrodes was verified by both optical microscopy for live cells and SEM analysis of fixed cells before and after the recording, respectively. A first group of devices (*n* = 3) was found to be characterized by a coverage of cells localized on the electrodes, like shown by the SEM images (left and central panels) in **Figure** **5**A. These cells adhered to the arrays retaining their typical morphology, already discussed in Section 3.3, and forming mostly interconnected chains along the electrodes. A detailed observation (right panel of Figure 5A) reveals a close contact of both body and projections with the Au/SiNWs without evidence of either cell membrane piercing or perforation. Figure 5B shows a representative temporal response recorded from electrodes of the NW_MEA with cells, while in Figure S2C, Supporting Information, typical voltage traces recorded from NW_MEA without cells and Au_MEA with cells are shown for comparison.

Time 0 represents the start of the recordings of the cell activity after the replacement of the culture medium with the HEPES solution without calcium (Ca^2+^ concentration = 0 mm). Figure 5B shows a noisy signal, obtained at Ca^2+^ concentration = 0 mm, with a RMS amplitude of 7.2 µV and the occurrence of sporadic and weak peaks. By elevating the external Ca^2+^ content we observe an increasing of the spike rate and amplitude as also evident in the waterflow plot of Figure 5C showing the segments of the trace in Figure 5B for different Ca^2+^ concentrations and selected at 2 min after the Ca^2+^ addition. On the other hand, the negligible signals observed in the case of the NW_MEA without cells and Au_MEA with cells (Figure S2, Supporting Information) indicate that the recorded spikes in Figure 5B are related to the spontaneous electrical activity of the cells grown on the Au/SiNWs electrode. Figure 5D shows a representative spike, recorded at 20 mm of extracellular Ca^2+^ concentration: the spike shape resembles the typical waveform of an intracellular AP of AtT‐20 cells^[^ ^42^, ^43^ ^]^ by suggesting a direct access of the NWs to the intracellular space.

The fact that the recording capability of the NW_MEA cannot simply be attributed to a decreased electrode impedance, but to an intimate contact between cells and NWs and a possible natural membrane penetration of the NWs is further corroborated by the comparative analysis performed with planar platinum MEA,Pt_MEA, as shown in Section S2, Supporting Information. We observed indeed that although the Pt electrode impedance was comparable to that of Au/SiNWs electrodes (Figure S2A, Supporting Information) and the AtT‐20 cells abundantly covered the Pt_MEA (Figure S2B, right panel, Supporting Information), negligible signals were recorded by the Pt_MEA. Very recent studies demonstrate that the tight adhesion and high local deformation of cells in contact with nanostructures, especially NWs,^[^ ^6^, ^63^, ^64^, ^65^ ^]^ can promote the permeabilization of lipids at the plasma membrane, providing intracellular access to the cells. In particular, membrane penetration will occur when a NW generates sufficient mechanical tension within the lipid bilayer to cause rupture, which may or may not pierce the cortical cytoskeleton. This mechanism is highly dependent on NW geometry and cell rigidity, and the local rupture of the membrane allows to perform opto‐/electro‐poration‐free intracellular recording from cell networks.^[^ ^25^, ^26^ ^]^

**Figure 6:**
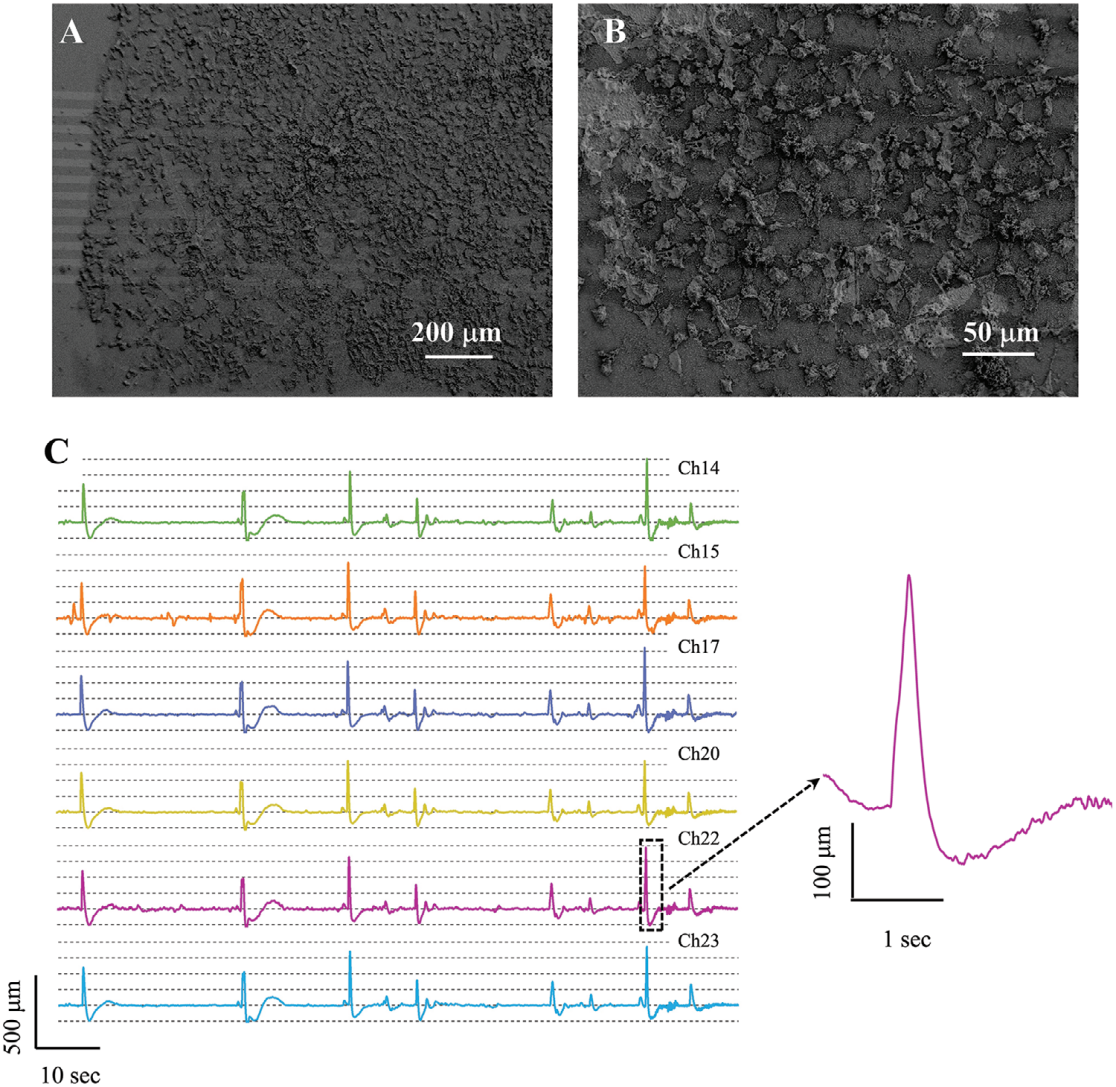
A) SEM images of a dense coverage of AtT‐20 cells on Au/SiNWs. B) Waterfall plot of the traces, recorded at Ca^2+^=50 mm, across six different channels (Chs). C) Zoom‐in view of a single spike recorded from channel Ch 22.

A summary of the number of events, amplitude (*V*_max_), and duration (*τ* = duration at 50% of *V*_max_) of the spikes as function of the calcium content are shown in **Figure** **6**E,F,G, respectively. The number of events were counted by considering only those spikes occurring during the whole activity recorded in (B) with *V*_max_ > 20 µV, that is about three times the RMS value obtained at Ca^2+^ concentration = 0 mm. We clearly observe an increase of the events with the extracellular Ca^2+^ concentration. The mean value of *V*_max_ slightly increases with Ca^2+^ by varying from mean 40 µV at 2 mm up to 60 µV at 50 mm, *τ* remains roughly constant with a mean value ranging between 150 and 180 ms. Our data are in line with the observations made using patch clamp technique on single AtT‐20 cells.^[^ ^43^, ^62^ ^]^

A second group of samples (*n* = 3) was characterized by a dense and connected network of AtT‐20 cells spreading out across the NW_MEA area as illustrated by the SEM images of Figure 6A. For this typology of samples, we observed that almost all channels exhibited synchronous signals for high extracellular Ca^2+^ content as shown by the waterfall plot in Figure 6B which plots representative recordings for six channels at 2 min following the administration of Ca^2+^ = 50 mm. We note the occurrence of synchronous peaks, whose waveform is still similar to the typical shape of an intracellular AP (Figure 6C), with amplitudes of several hundreds of microvolts and a duration of about 200–300 ms. The synchronicity of the signals cannot be attributed to shorted electrodes because impedance measurements of single electrodes were taken prior to seeding the cells. In addition, evident variations between the peaks’ intensity and related ratio as well as the occurrence of different low‐intensity features across the channels confirm non‐shorted electrodes. Then the observed spike synchronicity originated from networked AtT‐20 cell population. This phenomenon shows an impressive similarity to the synchronized activity observed in neuronal cultures with MEAs,^[^ ^26^, ^66^, ^67^ ^]^ whose origin is still unclear but related to collective dynamics of neurons. Thus, similarly to the neuron cultures on MEA, the synchronized AtT‐20 cells’ signals could be related to a coordinated activity of the connected AtT‐20 cells in the network, stimulated by the addition of a high dose of extracellular calcium.

On the opposite, if we consider the AtT‐20 cell population in Figure 6A,B as an ensemble of disconnected cells generating spontaneous APs, these cells could be depicted as intrinsic oscillators. Thus the synchronization could be the result of the coupling of single oscillators induced by the high extracellular Ca^2+^.^[^ ^67^ ^]^ Anyway the origin of the synchronized electrical activity observed in the second group of samples and the behavior of the first group of samples deserve to be deeply investigated, but this is beyond the scope of the present work which provides proof of concept for the potential of NW_MEA to report the electrical activity of electrogenic cells generating low amplitude APs.

## 4 Conclusion

In summary, our proposed approach has made possible to record by MEA technique for the first time, to the best of our knowledge, the weak electrical signals generated by a population of pituitary corticotrope cells. The ultra‐sensitivity of our MEA based on disordered SiNWs was demonstrated by the recording of signals in AtT‐20 cell populations exposed to high extracellular Ca^2+^ contents. Understanding the physiological role and significance of the observed signaling requires more detailed investigations, but the proposed NW_MEA introduces significant advances compared to state‐of‐the‐art microelectrodes and extend the usefulness of the MEA recordings to excitable cells characterized by very weak AP signals. Consequently, they pave the way toward the investigation of the electrical activity generated by the cells of the pituitary gland, which is crucial in controlling several essential human functions and our response to emotional and physical stress. The facile, high‐throughput and low‐cost fabrication, as well as the possibility to use glass or flexible substrates make the proposed NW_MEA a versatile tool suitable for electrophysiological drug screening, disease study and for in vivo recordings.

## Conflict of Interest

The authors declare no conflict of interest.

## Author Contributions

F.M. and L.M. contributed equally to this work. A.C. conceived and led all aspects of the project. F.M. and L.M. designed and developed the NW_MEA. F.M. developed the acquisition board. I.L. fabricated the NW_MEA. I.L. and J.I.D.R.D.V performed electrochemical characterization. A.C. performed the SEM imaging. M.L., A.S., and A.L. performed the cellular cultures and the viability/immunofluorescence/mRNA analysis. F.M., L.M., A.C., A.S., and M.L. performed electrophysiological recording. F.M. and A.C. performed the data analysis. V.M. participated to the data analysis. A.C. wrote the manuscript. All authors discussed the results and contributed to the manuscript writing and reviewing.

## Supporting information

Supporting Information

## Data Availability

The data that support the findings of this study are available from the corresponding author upon reasonable request.
