# Supplementary material for: Revealing Low Amplitude Signals of Neuroendocrine Cells through Disordered Silicon Nanowires‐Based Microelectrode Array
Source: Adv Sci (Weinh). 2023 Jun 25;10(24):2301925. doi: 10.1002/advs.202301925 (PMC10460871; doi:10.1002/advs.202301925)
Supplement: Supplementary file 1 — Supporting Information [file ADVS-10-2301925-s001.pdf]

ADVANCED SCIENCE
Open Access

Supporting Information

for *Adv. Sci.*, DOI 10.1002/advs.202301925

Revealing Low Amplitude Signals of Neuroendocrine Cells through Disordered Silicon Nanowires-Based Microelectrode Array

Francesco Maita, Luca Maiolo, Ivano Lucarini, Josè Ignacio Del Rio De Vicente, Antonio Sciortino, Mario Ledda, Valentina Mussi, Antonella Lisi and Annalisa Convertino**

Supporting Information

Revealing low amplitude signals of neuroendocrine cells through disordered silicon nanowires based microelectrode array

Francesco Maita†, Luca Maiolo †, Ivano Lucarini· José Ignacio Del Rio De Vicente, Antonio Sciortino, Mario Ledda, Valentina Mussi, Antonella Lisi, Annalisa Convertino**

† These authors contributed equally

E-mail: mario.ledda@cnr.it

E-mail: annalisa.convertino@cnr.it

S1. Viability of AtT-20 cells in high extracellular calcium environment

During the recording of the AtT-20 cells' electrical activity, the concentration of extracellular Ca^{2+} was tuned from 0 mM (HEPES buffer without calcium) up to 50 mM over a period of 36 min. To study the viability of the AtT-20 cells under those conditions we performed mRNA expression analysis of neurofilament heavy chain (NfH), class III beta-tubulin (TuJ1) and beta-actin (β-ACT), that are involved in cell motility, structure, and integrity. As controls we used: *i)* AtT-20 cells on a Petri dish, kept in their culture media for 36 min (CTRL1) without any external perturbation, and *ii)* AtT-20 cells on a Petri dish in HEPES buffer by increasing the extracellular Ca^{2+} content (from 0 up to 50 mM) over a period of 36 min (CTRL2) with the very same modality used in the recording experiments. **Figure S1**A shows no statistically significant differences between the measured mRNA markers of the cells on Au/SiNWs, exposed to high Ca^{2+} concentrations, (gray histograms) compared to the controls (blue and orange histograms for CTRL1 and CTR2, respectively). In addition, the optical images in Figure S1B, representing the very same AtT-20 cell population on Au/SiNWs in HEPES with Ca^{2+} = 0 mM, 30 mM, and 50 mM, do not show any morphological modification that could suggest cells suffering or death. Collectively these results indicate that the investigated external Ca^{2+} concentration range did not show cytotoxicity towards the AtT-20 cell populations over a period of exposure as long as 36 min.

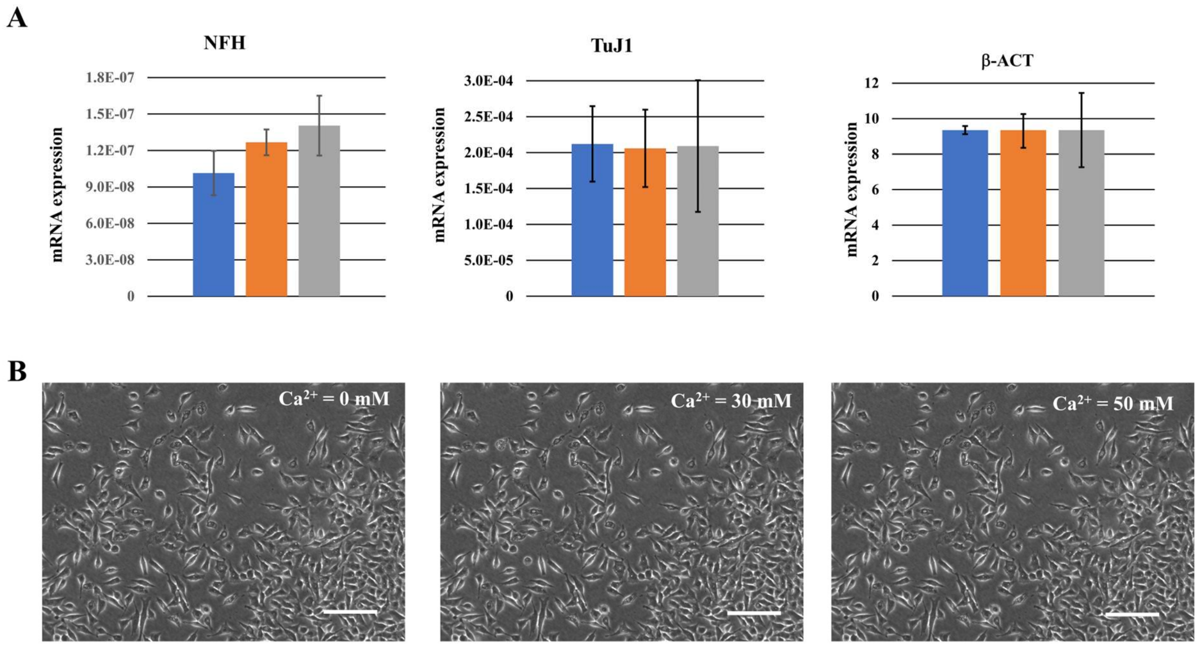

Figure S1. Effects of the high extracellular calcium concentrations, tuned from 0 mM up to 50 mM, on the viability of the AtT-20 cells over an exposure period of 36 min. (A) RT-qPCR analysis of NFH, TuJ1 and β-ACT markers for *i)* AtT-20 cells cultured on Au/SiNWs mat after exposure to high Ca^{2+} concentrations (gray histograms), *ii)* AtT-20 cells cultured on a Petri dish and kept in their culture media for 36 min without any external perturbation (CTRL1, blue histograms), and *iii)* AtT-20 cells plated on a Petri dish and exposed to high Ca^{2+} concentrations (CTRL2, orange histograms). (B) The morphology of the AtT-20 cells does not change as shown by the optical images of the culture for Ca^{2+} = 0 mM, 30 mM, and 50 mM. The images were kept 3 min after adding calcium. Scale bar 100 μm.

S2. Comparison between the extracellular recordings performed with NW_MEA and planar MEA

To verify that the spikes recorded by NW_MEA were related to the electrical activity of the cells we performed extracellular recording by using planar gold MEA (Au_MEA), characterized by the same electrode number, size and configuration of the NW_MEA. To define further the characteristics of the device, we used also planar platinum MEA (Pt_MEA) because of a better electrode impedance of Pt with respect to Au.[1,2] In Figure S2A we observe indeed that the mean electrochemical impedance of the planar Pt electrodes (blue symbols) at the frequencies of 30 Hz, 100 Hz, 1k Hz and 5k Hz is lower than that of the planar Au and very close to that of Au/SiNWs electrodes. We acquired the voltage vs. time traces in the case of the AtT-20 cells cultured on both Au and Pt MEAs (in Figure S2B the AtT-20 cell population on Au_MEA and Pt_MEA, left and right panels respectively). Figure S2C shows the raw data of

the representative voltage vs. time traces recorded from one selected electrode of NW_MEA (black), Au_MEA (yellow) and Pt_MEA (blue) with cells.

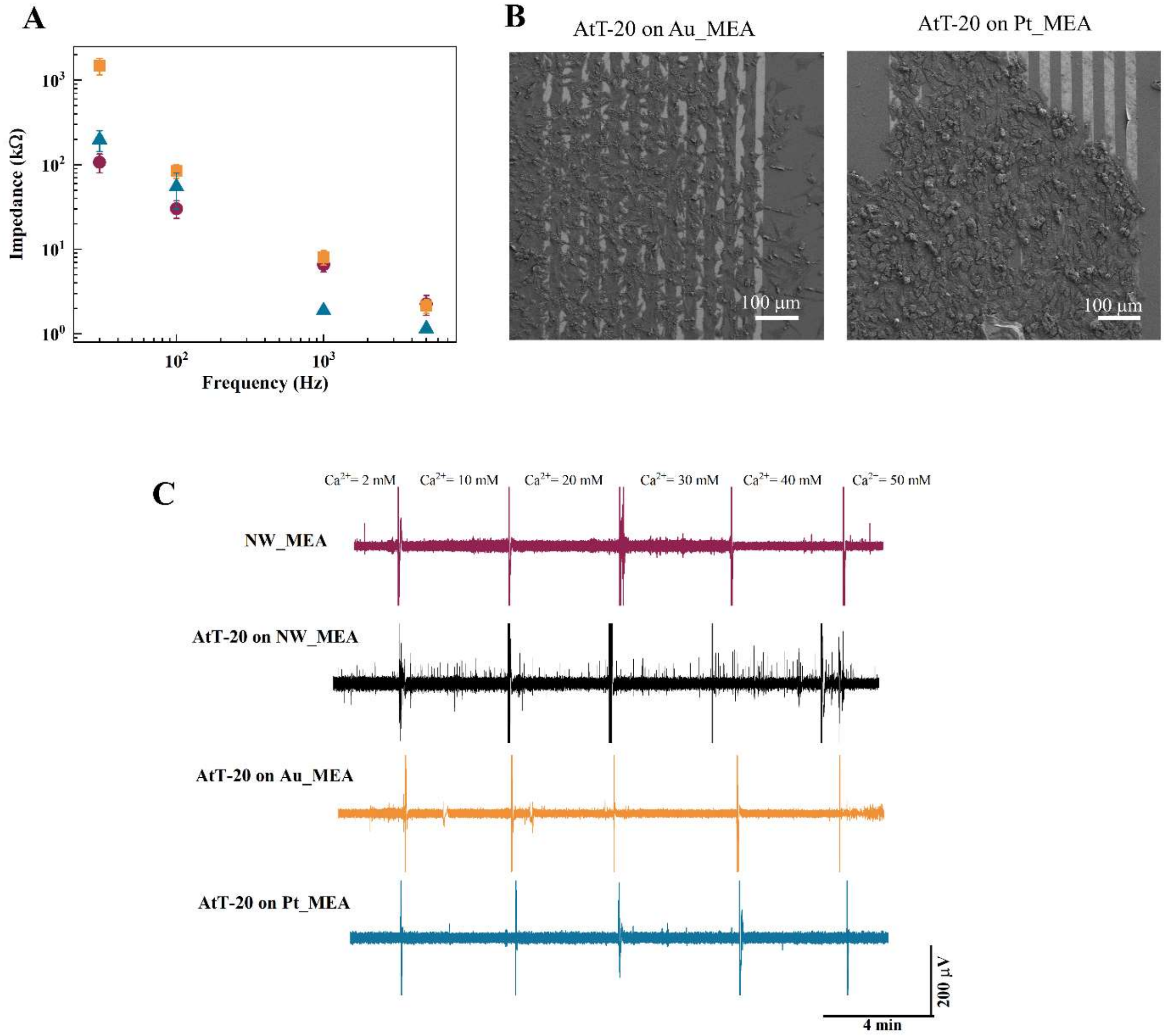

Figure S2: (A) Average electrochemical impedance of the Au/SiNWs (red symbols), Au (yellow symbols) and Pt (blue symbols) planar electrodes at the frequencies of 30 Hz, 100 Hz, 1k Hz and 5k Hz (N=36). The value of the standard deviations is indicated by the error bars. (B) SEM images of AtT-20 cells on Au (left panel) and Pt (right panel) planar MEAs. (C) Raw data of the representative voltage vs. time traces recorded from one selected electrode of NW_MEA (black), Au (yellow) and Pt (blue) planar MEAs with AtT-20cells. The upper trace (red) is obtained by one representative electrode of NW_MEA without cells. The four traces were recorded in the very same conditions by tuning the extracellular Ca^{2+} concentration in the range of 2-50 mM. The traces are temporally aligned.

The upper trace in red is obtained by one representative electrode of NW_MEA without cells. The four traces were recorded in the very same conditions, i.e. after 4 min at Ca^{2+} = 0 mM

(HEPES without calcium) the extracellular Ca^{2+} concentration was increased each 4 min from 2 mM up to 50 mM. The five large and periodic voltage peaks observed in all the traces were caused by the Ca^{2+} addition in the extracellular medium and have been used to temporally align the signals from the different devices. A negligible number of spikes was observed in the case of Au_MEA with cells and NW_MEA without cells by suggesting that the signals registered from NW_MEA with cells can be attributed to the electrical activity of the AtT-20 cells. Furthermore, we note that there was no signal from the Pt_MEA cells too, although the Pt electrode impedance was comparable to that of Au/SiNWs electrodes (Figure S2A) and the AtT-20 cells abundantly covered the Pt_MEA (Figure S2B right panel). Collectively these results indicate that the recording capability of the NW_MEA cannot simply be attributed to a decreased electrode impedance, due to the surface nanostrcturing with NWs, but to a closer contact between NWs and cells and a possible natural membrane penetration of the NWs.

References

[1]] C. Li, R. K. Narayan, P. M. Wu, N. Rajan, Z. Wu, N. Mehan, E. V. Golanov, C. H. Ahn, J. A. Hartings JA. *J Neural Eng*. **2016** 13:016008. doi: 10.1088/1741-2560/13/1/016008.

[2] G. H. Kim, K. Kim, E. Lee, R. An, W. Choi, G. Lim, J. H. Shin. *Materials* **2018,** 11, 1995. DOI: 10.3390/ma11101995
